# Analysing sentiment change detection of Covid-19 tweets

**DOI:** 10.1007/s00521-023-08662-2

**Published:** 2023-05-31

**Authors:** Panagiotis C. Theocharopoulos, Anastasia Tsoukala, Spiros V. Georgakopoulos, Sotiris K. Tasoulis, Vassilis P. Plagianakos

**Affiliations:** 1 Department of Computer Science and Biomedical Informatics, University of Thessaly, Lamia, Greece; 2 Department of Mathematics, University of Thessaly, Lamia, Greece

**Keywords:** Sentiment change detection, Covid-19 tweets, BERT, PELT

## Abstract

The Covid-19 pandemic made a significant impact on society, including the widespread implementation of lockdowns to prevent the spread of the virus. This measure led to a decrease in face-to-face social interactions and, as an equivalent, an increase in the use of social media platforms, such as Twitter. As part of Industry 4.0, sentiment analysis can be exploited to study public attitudes toward future pandemics and sociopolitical situations in general. This work presents an analysis framework by applying a combination of natural language processing techniques and machine learning algorithms to classify the sentiment of each tweet as positive, or negative. Through extensive experimentation, we expose the ideal model for this task and, subsequently, utilize sentiment predictions to perform time series analysis over the course of the pandemic. In addition, a change point detection algorithm was applied in order to identify the turning points in public attitudes toward the pandemic, which were validated by cross-referencing the news report at that particular period of time. Finally, we study the relationship between sentiment trends on social media and, news coverage of the pandemic, providing insights into the public’s perception of the pandemic and its influence on the news.

## Introduction

The global spread of the highly infectious respiratory disease caused by the coronavirus SARS-CoV-2 declared as Covid-19 (coronavirus disease 2019) by the World Health Organization (WHO), ignited a pandemic on a worldwide scale, which affected heavily worldwide and nationwide the well-established social, economic and political systems. Governments around the world took immediate action to counteract the contagiousness of the virus, and along with the guidance of WHO, a variety of measures were enforced. Among the measures implemented, lockdowns, quarantines, and social distancing rules were some of the strategies employed to slow the spread of the virus. These measures ended up having a major impact on daily life and disrupted many aspects of society, including work, education, and social gatherings. Also, many of the tools that were devolved, aimed to facilitate the medical personnel in their fight during this global health crisis, contributed crucially to further prevention of virus transmission.

One of the first attempts to combat the Covid-19 pandemic is through the development of contact tracing apps. These apps use Bluetooth technology to anonymously track the movements of people who have installed the app on their phones. If a person tests positive for Covid-19, the app can alert other users who may have come into close contact with that person, so that they can take appropriate steps to protect themselves and prevent the spread of the virus. However, the tools using AI techniques are fewer in comparison to the non-AI methods [1]. During the pandemic, AI-powered methods have been used in various fields against the pandemic, e.g. the analysis of medical images and the identification of patients with Covid-19 which helped the specialists to make faster and more accurate diagnoses [2]. Additionally, the researchers and health-related officials developed and used predictive models to track the spread of the virus and to forecast its spread [3]. Last but not least, the AI-powered methods helped in developing new drugs and therapies to treat Covid-19. Machine learning algorithms have been used to analyse the virus’s genetic sequence and identify potential targets for drugs [4].

Artificial intelligence (AI) has been used to analyse large amounts of text data, including microblogging platforms like Twitter, for tasks such as sentiment analysis and toxicity detection [5, 6]. AI tools have also been applied to social media posts and search queries in order to track the spread of the Covid-19 virus and monitor public sentiment [7]. Researchers from various fields have used Twitter as a source to study the impact of the pandemic on society, as it allows for easy aggregation and analysis of large amounts of tagged and specified data. By examining tweets, researchers have been able to identify patterns and trends related to the pandemic and develop strategies for combating its spread. For instance, some research has focused on the psychological effects of the pandemic on individuals [8] and others have investigated the economic impacts of lockdowns and other measures implemented to contain the virus [9].

Measuring health policy is a well-known problem, and there is no universal measure or strategy since it is related to the nature and type of health problem that legislation, regulation, and common practices are trying to address [10–12]. The common timeframe for evaluating these strategies may vary from months to years. Additionally, the policy window can also vary, and their effects may not be immediately reflected in society [13]. A tool that can indicate the major changes that have occurred, and their relationship with the competent institutions and public domains, would provide significant insights into health policies.

In this paper, we propose a sentiment analysis framework to study public opinion on a daily basis at Twitter for Covid-19, producing a time series and detecting significant changes among them. We assume that a significant worldwide event will be reflected in public opinion on Twitter messages, imposing a distributional change in the produced sentiment time series. By analysing the sentiment of tweets, social media posts and other forms of online communication, researchers could identify common themes and concerns among the general public. Furthermore, sentiment analysis could also be used to study how people feel about potential vaccines and other treatments for future pandemics. To the best of our knowledge, there is no other study that associates the sentiment from tweets to detect significant Covid-19 events. The rest of the study has been conducted in five sections. Section 2 represents related studies using various methods for text classification. Section 3 describes the dataset and the proposed methodology. Section 4 presents the performance of each method, followed by the discussion of the results in Sect. 5. Finally, Sect. 6 includes the conclusion of the study and future directions.

## Background

A significant amount of research on sentiment analysis of tweets related to the Covid-19 pandemic has been conducted. A study by Wisesty et al. [14] used Twitter data to evaluate three different methods for performing sentiment analysis. These methods included the vector space model with support vector machine, word embedding with long short-term memory, and BERT. The results showed that BERT was the most effective method, achieving a weighted F1-score of 0.85. Another study showed that social media plays a significant role in shaping public sentiment, particularly during times of crisis such as the Covid-19 pandemic. The study found that negative sentiment on social media can have a major impact on public opinion, and can even influence policy decisions. This suggests that social media platforms have a responsibility to moderate their content and prevent the spread of misinformation. By proactively addressing negative sentiment and providing accurate information, social media platforms can help mitigate the negative effects of misinformation on public health. Additionally, public health officials should be prepared to actively engage with social media users in order to counter negative sentiment and promote accurate information. The findings of the study were based on using Word2Vec, term frequency-inverse document frequency (TF-IDF), GloVe, BERT and a distilled version of BERT (DistilBERT) [15]. Researchers also used machine learning and lexicon analysis to analyse tweet sentiment in the USA during the early stages of the Covid-19 pandemic. The study used a dataset of 11, 858 tweets collected from January 30 to May 10, 2020. The study applied the TextBlob lexicon to label each tweet as positive, negative, or neutral. The results of the study showed that the gradient boosting machine paired with the TF-IDF feature technique performed the best, achieving an accuracy of $96\%$ [16]. Similarly, the [17] used Twitter data to analyse the sentiments of users regarding Covid-19. The dataset consisted of tweets from nine states in the USA over a period of 15 days in April 2020. The researchers labelled the tweets into three categories based on sentiment: positive, negative, and neutral. They then used machine learning and deep learning approaches to classify the sentiments of the tweets. The researchers also compared different methods, including traditional BoW and TF-IDF, as well as different word embedding schemes such as Word2Vec and GloVe. They also compared the number of Covid-19 infection cases with the number of Covid-19-related tweets during the pandemic.

Additionally, other studies focused on the sentiment over the Covid-19 vaccines. In one of the earliest published papers on vaccine-related tweets, researchers used a comprehensive latent Dirichlet allocation (LDA) topic modelling approach to analyse the subjectivity of the tweets and the use of war-related vocabulary in figurative framing. The study aimed to understand how sentiment towards vaccines was represented in the tweets [18]. Moreover, the study of Cotfas et al. [19] used a variety of techniques to analyse vaccine-related tweets and compare the results to real-world events. The specific methods mentioned, including bag-of-words (BoW) representation, word embedding with machine learning, word embedding with deep learning, and BERT, are all common techniques used in natural language processing and text analysis. By comparing the results of these techniques to actual events, the authors of the study were able to draw conclusions about how well the tweets reflected real-world trends. In another study, researchers used the BERT model to develop a detection system for vaccine misinformation on Twitter. They compared the performance of this system to two other popular machine learning models, LSTM and XGBoost, and found that BERT had the highest precision test score of 0.98 [20]. The [21] presents the findings of a study on Covid-19 related tweets, specifically focusing on the Omicron variant of SARS-CoV-2. The study analysed 12, 028 tweets and examined several characteristics including sentiment, language, source, type, and embedded URLs. The results showed that $50.5\%$ of the tweets had a neutral sentiment and $65.9\%$ were posted in English. In addition, the [22] proposed a method for classifying tweets using five different machine learning models: logistic regression, random forest classifier, multinomial NB classifier, support vector machine, and decision tree classifier. The researchers evaluated the performance of these models using metrics such as precision, recall, f1-score, and support and also presented the results in the form of a confusion matrix, accuracy, precision, and receiver operating characteristic (ROC) graphs.

One of the main challenges in sentiment analysis is tracking changes in users’ sentiment over time. Tracking changes in users’ sentiment over time is a complex task in sentiment analysis due to the contextual dynamics, subjectivity and variability of sentiment, data scarcity, noise and ambiguity in text data, and scalability requirements. Addressing these challenges requires advanced techniques, algorithms, and models that can accurately capture and analyse changes in sentiment while considering the complexities of language, human emotions, and data characteristics [23–26]. The [27] discusses the use of sentiment analysis on Japanese Twitter data to understand the relationship between social media reactions and the progression of the Covid-19 epidemic in Japan. The analysis showed that there is a repetitive relationship between the relevant reactions on Twitter and the progression of Covid-19. A deep neural network model was proposed to capture this relationship and predict the future trend of Covid-19 progression, which could then be used to set up a susceptible-exposed-infected-recovered model for simulating potential future cases of Covid-19. The experiments conducted to evaluate the potential of using tweets to support the prediction of how an epidemic will progress demonstrated the value of using epidemic-related social media data. The findings suggest that there is a relationship between user reactions on social media, particularly Twitter, and epidemic progression, which can be useful in fighting pandemics. Saito et al. [28] describes a model that utilizes transformer-based neural network models to analyse the relationship between social media sentiment and the progression of the Covid-19 pandemic in major cities such as New York, Los Angeles, and Chicago. The model was found to be effective when tested in these cities. The time series analysis of social sentiment in these cities over a two-year period, including the pre-pandemic period and the current "new normal," demonstrated the presence of similar patterns in the three cities. The authors propose that the methods presented in their paper could be applied to the analysis of various emergencies and serve as a policy support tool in addition to traditional surveys. Overall, these studies highlight the importance of using advanced techniques such as machine learning and natural language processing in analysing the sentiment of tweets related to the Covid-19 pandemic, as well as the need to consider the source and topic of the tweets in order to accurately understand the sentiment expressed. Lastly, novel frameworks have been proposed for tracking the user’s sentiment over time. The study [29] describes a method for real-time detection of changes in sentiment using open-source tools. The approach involves collecting tweets in real time, using a lexicon approach to characterize sentiment, and using control charts to detect changes in sentiment without requiring historical information. The goal of this method is to identify fake news or propaganda efforts in their early stages by monitoring threads on a large scale. The approach has been tested in real-time analysis and has shown the ability to detect meaningful changes in sentiment over the lifetime of a hashtag. The study [30] aims to monitor and analyse depression trends on Twitter during the COVID-19 pandemic. The study proposes a novel approach to automatically create a large-scale depression user dataset using transformer-based deep learning language models. It also investigates the importance of psychological text features in depression classification. Furthermore, the study utilizes the developed model to track the fluctuation of depression levels among different user groups as the pandemic progresses. To identify trend changes, the study aggregates past tweets of individuals within a specific time frame and employs the depression classification model to categorize them as either depression or non-depression users. By comparing the depression level trends of identified depression and non-depression groups, as well as groups from different geolocations, over time, the study reveals that the non-depression group’s depression level increased earlier than that of the depression group. This finding is supported by relevant psychological theories and LDA topics extracted from key time points. What sets our study apart from other research is its comprehensive sentiment analysis approach. We combine NLP techniques and ML algorithms to classify the sentiment of each tweet as positive or negative. We then leverage sentiment predictions to conduct a time series analysis throughout the course of the COVID-19 pandemic. Additionally, we employ a change point detection algorithm to identify crucial shifts in public attitudes towards the pandemic, which are validated by cross-referencing news reports during those specific time periods. Finally, we explore the relationship between sentiment trends on social media and news coverage of the pandemic, providing valuable insights into the public’s perception of the pandemic and its influence on the news. These nuanced aspects of sentiment detection contribute to a better understanding of public attitudes towards pandemics and sociopolitical situations in general, which can inform decision-making processes in various domains, including healthcare and politics.

## Proposed methodology

**Fig. 1:**
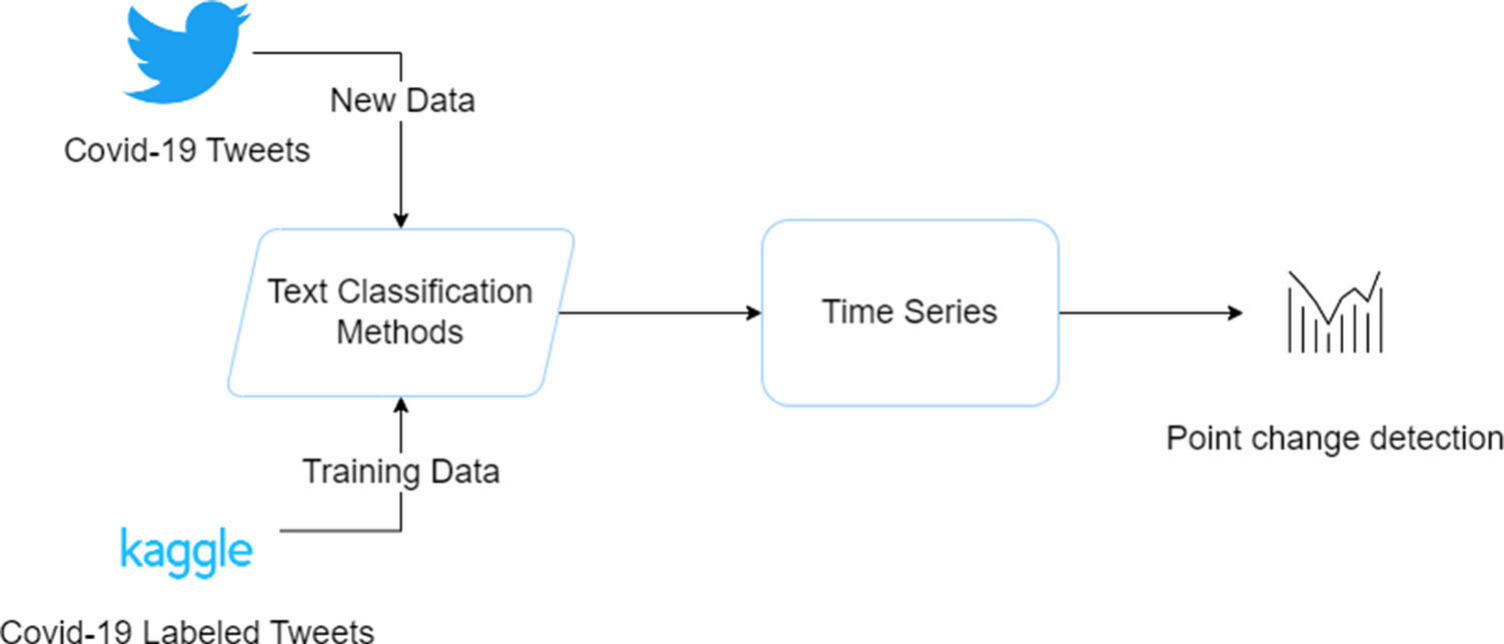
Flowchart of the proposed methodology

This study proposes a sentiment analysis framework for analysing social media text, in this case, related to Covid-19 and vaccination. The framework is designed to identify key points in time when significant Covid-19 events have occurred worldwide. The effectiveness of the proposed framework is then evaluated by examining its ability to align with news reports covering the same time period. Figure 1 provides the flowchart of the proposed methodology by displaying the core acts as follows:

- Collection of labelled on sentiment Covid-19 tweets;
- Fine-tuned BERT model;
- Hydration of new Covid-19 related tweets, within the year 2021;
- Creation of average sentiment time series per day;
- Detection of breakpoints on the time series;

### Dataset description

The data used to train and validate the methods were obtained from a Kaggle competition, which contained 5000 sentimental Covid-19 tweets [31] and from this point onward we will refer to it as the training dataset. The Kaggle dataset used for our study consisted of 11 labels, with many tweets having more than one label, resulting in a total of 256 unique label combinations. To simplify our analysis, we only considered tweets with a single label and classified them as either positive or negative based on specific labels. Specifically, we classified tweets with labels such as Optimistic, Thankful, Empathetic, Surprise, and Joking as positive, and tweets with labels such as Pessimistic, Anxious, Sad, Annoyed, and Denial as negative. The “Official report” label was excluded from our analysis, as our focus was on capturing people’s sentiments. Thus, the total tweets used for training the models were 1375 tweets. Furthermore, to capture peoples’ sentiment over Covid-19 and its vaccines, we used Twitter because of its popularity. The data were gathered from the IEEE data port [32]. However, the original dataset contained only the tweet’s ID, due to Twitter’s new policy to hide the user profile and sensitive information. In order to retrieve the tweet’s complete information, we used the Twitter Application Program Interface (API) by searching the tweet’s ID, a method called hydrating. In order to ensure that the analysis is based on high-quality data, we had to perform a text-cleaning procedure. The cleaning procedure involved:

- Removing special characters such as hashtags, emojis, and URLs,
- Removing stop words, such as “the”, “and”, “of”, etc. because they do not convey meaningful information,
- Removing duplicates because it is common for tweets to be re-tweeted or shared multiple times but also to avoid having multiple tweets from bots,
- Normalizing text involves converting all text to a consistent format, such as lowercase, to make it easier to process and analyse

**Fig. 2:**
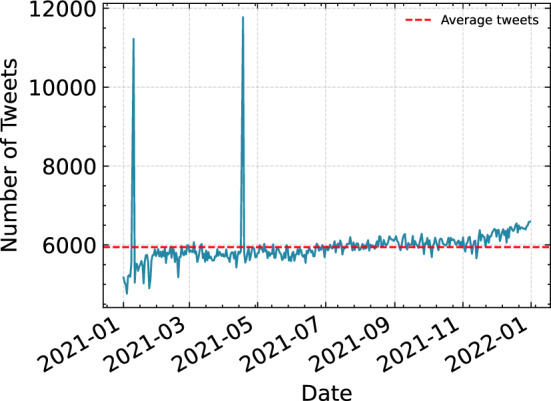
Tweets per day

Finally, the obtained data contain 2,157,747 in total tweets between January 01, 2021, and December 31, 2021, and from this point onward, we will refer to it as the tweet’s dataset. Figure 2 displays the obtained tweets per day with an average of $\sim 5,944$ tweets/day.

### Sentiment time series

Different methods have been applied for identifying the sentiment of users in microblogging, as described in Sect. 2. In our previous work [33], we used a transformer-based machine learning technique, called bidirectional encoder representations from transformers (BERT) [34]. BERT uses a bidirectional representation, meaning that the words can see themselves in comparison with the unidirectional context where the representations are building incrementally. Thus, the model can recognize the total sequence of words and understand its content [35]. The model’s training uses a masked language model (MLM) objective. This approach masks out 15% of the input words. These tokens are $80\%$ of the time replaced with a “MASK” token, 10% with a random word and 10% by keeping the same word. Bert has two models, the base model with 12 encoders, which we used in our experiment, and the large model with 64 encoders.

**Fig. 3:**
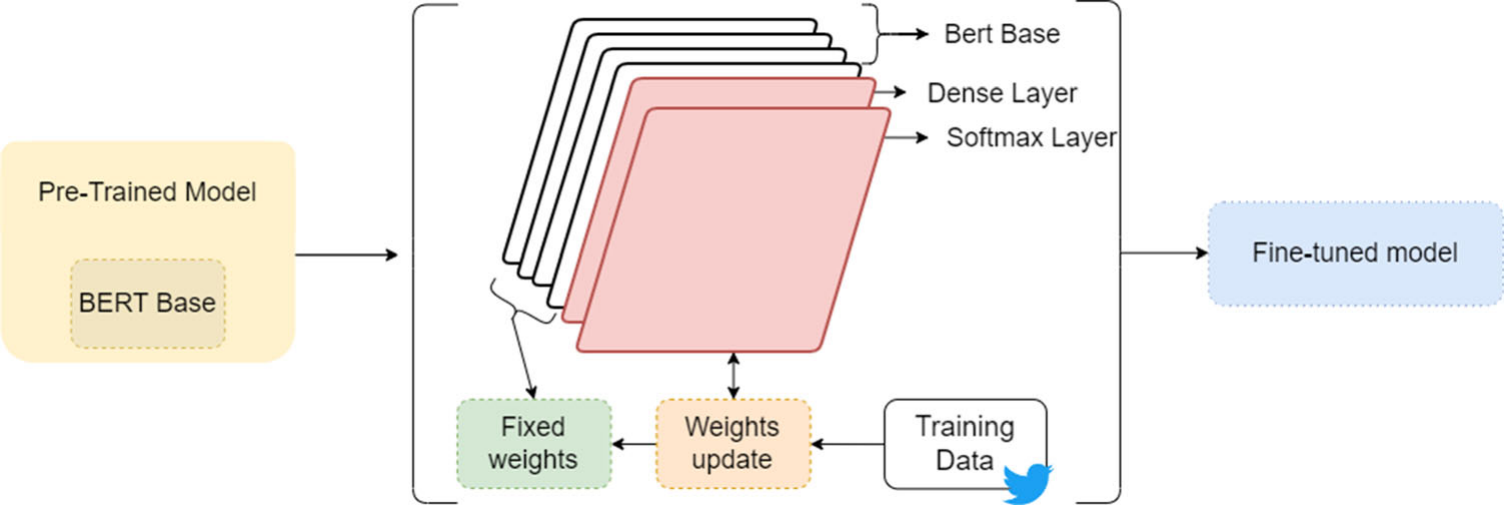
Schematic summary of the adjusted architecture

In addition, the model can perform a *Next Sentence Prediction*. The model pre-train text pairs together; thus, it learns the relationships between sentences and can predict whether a sentence has actually proceeded from the original sentence or a random one. The BERT’s input is comprised of token embeddings, segment embeddings and positional embeddings. The word tokenization has been performed by the BERT tokenizer. The tokenizer uses the word-piece tokenizer concept, which breaks some words into sub-words or into word pieces if the word can be broken into multiple tokens [33–35]. We modified the pre-trained model by adding a dense layer and a softmax layer to it in order to fine-tune the model. During the fine-tuning of the model, we updated the weights of the pre-trained BERT model on the training dataset. The weights are adjusted to minimize the error between the predicted output and the true output. Updating the weights during fine-tuning allows the model to learn and adapt to the specific characteristics of the training dataset, which can improve the model’s performance on that dataset. The adjusted architecture is illustrated in Fig. 3.

### Point change detection

Time series analysis has become more and more important in a spectrum of areas. Change point detection can identify sudden changes in the attributes of the time series [36]. It uses two different categories, online methods and offline methods. The major point of the online method is to detect a change as soon as it happens in real-time, whereas the offline method detects the changes when all the data samples are received, as a retrospective view [37]. Furthermore, the offline method can be applied when the number of change points is unknown [38]. In order to identify the turning points in people’s sentiments, we used the pruned exact linear time (PELT). It is an algorithm used to detect changes in the mean of a time series. It is called "pruned" because it uses a pruning technique to reduce the amount of computation needed to detect changes, and "exact linear time" because it has a computational complexity of *O*(*n*) in the number of data points, where n is the length of the time series. This means that the running time of the algorithm grows linearly with the size of the input data. The PELT algorithm works by dividing the time series into a series of contiguous segments, where each segment has a constant mean. The algorithm then searches for the "optimal" set of breaks in the time series that minimizes the sum of the squares of the residuals (i.e., the difference between the observed values and the mean of the segment). One advantage of the PELT algorithm is that it is able to detect changes in the mean of the time series with high accuracy, even when the data is noisy or the changes are small. It is also relatively fast, making it a useful tool for online change point detection, where the data is streamed in real-time and changes need to be detected as quickly as possible. There are some limitations to the PELT algorithm, however. It is sensitive to the choice of the penalty parameter, which controls the trade-off between the number of change points detected and the fit of the model to the data. Finally, the PELT algorithm does not provide any information about the nature or magnitude of the change that has occurred. It only indicates that a change has occurred, and it is up to the user to investigate the data further to determine the cause of the change [37–39].

The cost function of the PELT algorithm can be calculated by

$$c(k) = \min \left( {\sum\limits_{{j = 1}}^{{m + 1}} c \left( {y_{{(t_{j} - 1) + 1:t_{j} }} } \right) + \alpha } \right)$$

where *C* is the cost function and $\alpha$ is the penalty. The penalty is a hyperparameter that controls the sparsity of the solution, (i.e. the number of change points detected in the data). A larger penalty value will result in fewer change points being detected. The algorithm works by iterating over all possible positions for the change point and calculating the cost of the data split at that position. The position with the minimum cost is chosen as the change point [37, 38].

## Experimental results

This section presents the results of how each method performed and captured the sentiment analysis on the Covid-19 tweet dataset. To evaluate the effectiveness of various sentiment analysis methods, we conducted a comparison using a range of techniques including the bag-of-words approach, the term frequency-inverse document frequency method with a support vector machine classifier [40], Word2Vec with a random forest algorithm [41], the BERT model, and a lexicon-based method called TextBlob.

The training dataset contains 891 negative and 484 positive cases. To address the issue of the imbalanced data, we calculated the class weights based on the formula:

$${\text{weight}}(c) = n_{{{\text{entries}}}} /\left( {n_{{{\text{classes}}}} *fd(c)} \right)$$

where $n\_entries$ is the total number of samples in the dataset, $n\_classes$ is the total number of classes, and *fd* is the frequency distribution of values that occurred [42]. The calculated weights have been applied to the loss function during the training of the model. The hardware used for the experiments contains an i7-10700K CPU, 128GB RAM, and an NVIDIA Titan X 12 GB GPU, running on an Ubuntu 20.04 OS. Table 1 provides the average results of each method after 100 independent iterations. To prevent overfitting, we performed an early trigger in our model by monitoring the train and validation loss. Once the validation loss reaches its minimum score and starts increasing, we stop the training and save the current model. The early stopping method has been utilized on a validation set, which constitutes 10% of the training data and is distinct from the data used for actual training. This approach allows the model’s performance to be assessed on unseen data. To mitigate the risk of falling into local minima, the experiments were conducted multiple times with significant overfitting, and it was observed through empirical analysis that early stopping was an appropriate technique to employ. From the results, we notice significantly similar accuracy scores between the BoW and TF-IDF methods. The TF-IDF method has a lower area under the receiver operating characteristic curve (AUC) 0.72 score in comparison to the BoW with an AUC score of 0.77. A further inspection of the data in the results table reveals that Word2Vec also performs similarly to the other aforementioned methods but with a lower AUC score of 0.69. The training data have a specific domain; thus, the BoW may work better than Word2Vec, since it may not be possible to find corresponding vectors from pre-trained word embedding models.

**Table 1:** Mean accuracy scores with a standard deviation of each method on the validation data

Method	Accuracy	Standard deviation
BERT	0.990	0.003
BoW	0.721	0.147
TF-IDF	0.726	0.124
Word2Vec	0.703	0.119

### Time series comparison

**Fig. 4:**
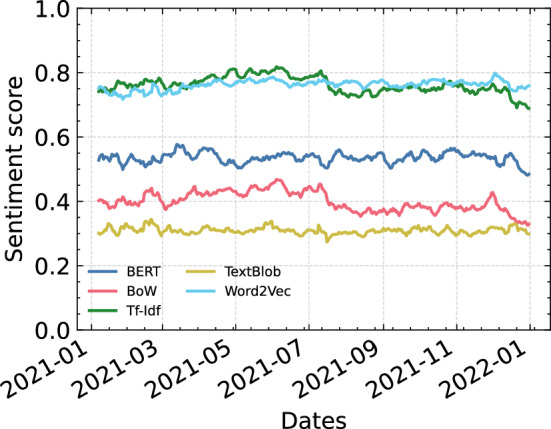
Sentiment time series of each method

After training the models, we calculated the average sentiment per day on the tweet’s data, leading to a sentiment time series of each method within the year 2021. Among the results, we additionally performed a time series by using a lexicon-based sentiment, TextBlob. The results of each method are set out in Fig. 4. The daily sentiment of each method has been determined by calculating the average probability of the tweets for each day. The score towards 0 presents negativity and towards 1 positivity. What stands out in the figure is that even though BoW and TF-IDF performed similarly, the sentiment scores are significantly different with $\sim 36\%$ difference. On the other hand, Word2vec and TF-IDF are closely together towards the positive axis with an average sentiment of $76\%$. On the contrary, TextBlob is closer to the negative axis with an average score of around $30\%$. BoW has average sentiment around $39\%$ and the sentiment analysis performed using BERT resulted in a score of $52\%$, which is around the neutral axis. This suggests that the analysis reflects the real sentiment of the users, as opposed to the average sentiment scores of the other methods and by prior studies such as [21], which showed that $50.5\%$ of the tweets had a neutral sentiment. A major advantage of transformers is the dynamic word embeddings while other methods, like Word2Vec or GloVe, are based on static word embeddings.

### Breakpoints

**Fig. 5:**
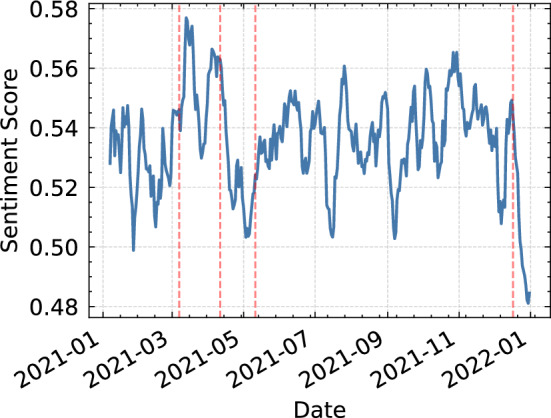
Breakpoints in BERT time series

Based on the previously described results, we further analyse the time series produced by the BERT model based on its overall better performance. Therefore, we applied the PELT method. The original penalty formula used in PELT detected multiple sentiment changes, but upon reviewing the outcomes, it was determined that the identified breakpoints were not completely accurate. To rectify this issue, adjustments were made to the sensitivity parameter of the penalty, resulting in changes to the average sentiment. The produced breakpoints are shown in Fig. 5 with timestamps on the following dates: 07/03/2021, 11/04/2021, 11/05/2021 and 17/12/2022.

**Fig. 6:**
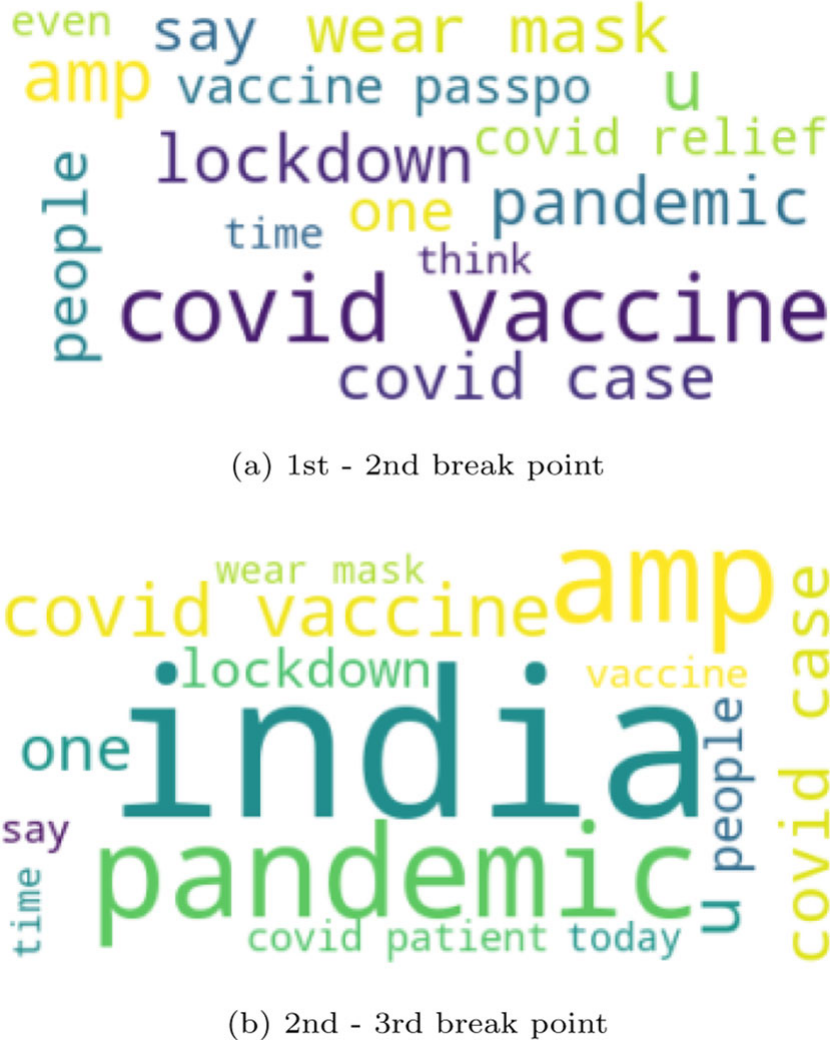
Top 15 words in each breakpoint

To further investigate the produced results, we produced a word cloud (Fig. 6) based on the tweets published tweets in each period. The word cloud method could help us identify insights on trends and patterns as a visual representation of the text. When a word appears bigger than the others, the more often is mentioned. Figure 6 shows the words mentioned within the area of the first and the second breakpoint compared with the time period of the second and third breakpoint. The results indicate the change in the discussed topics.

**Fig. 7:**
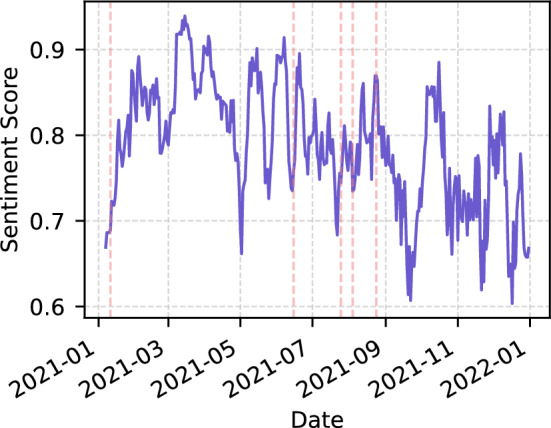
Breakpoints in AstraZeneca’s time series

We analysed tweets that mentioned the AstraZeneca/Oxford or Vaxzevria Covid-19 vaccine companies and used a change detection algorithm to track the sentiment about the vaccine over time. The results are shown in Fig. 7, which shows that there were significant changes in sentiment on the following dates: 11/01/2021, 15/06/2021, 25/07/2021, 04/08/2022 and 24/08/2022.

## Discussion

In 2021 the Covid-19 pandemic, many countries implemented lockdowns to prevent the further spread of the virus. As the citizens of the world experienced this uncommon situation, their way out to interact with other people was mainly the social media. The social media platforms’ traffic has gone beyond expectations. One of the most popular platforms is Twitter, with a diverse spectrum of users worldwide. Several reports have shown that social media users’ opinions could reflect the current sociopolitical situations in their area. Sentiment analysis on data collected from Twitter is considered one of the most commonly applied methods. It is a powerful tool for understanding emotions and opinions about a topic. One of the main objectives of this study was to use state-of-the-art methodologies for analysing people’s sentiments. As mentioned in Sect. 4, we performed five different approaches on the same data to capture the sentiment. Furthermore, the methodology applied in this study, after the training of the model, does not require high computational resources and shows low computational complexity.

Further analysis of the produced time series showed interesting results. Taking a closer look at Fig 2, on the 10/01/2021 we had an increase in the published tweets. The cross-reference with the media reports from this period showed that it overlaps with the time when the administration of the Covid-19 vaccines started. For example, on January 7th, 2021, the United Kingdom announced the approval of the vaccines from Pfizer/BioNTech and AstraZeneca [43]. As part of the general discussion related to vaccines, the BMJ journal published an article on the UK’s vaccination policy regarding doses [44]. It also coincides with a period when there was a huge spike in the number of deaths in the USA [45].

The point change detection led us to extend the analysis within the dates 07/03/2021 and 11/04/2021. A retrospection of that time span showed that the news focused mainly on the vaccination against the virus. The main topics concerned the vaccines’ side effects followed by the WHO official statement regarding AstraZeneca’s vaccine causing Thromboembolic events [46–48]. Another finding in the newsletter was the $\$1.9$ trillion relief law in the USA. These results seem to be consistent with the observations made in Fig. 6a.

If we now turn to the analysis within the timespan during the second and the third breakpoints (11/04/2021 - 11/05/2021), it is noticeable in Fig. 6b that the main discussion on Twitter has changed. The verification of the findings on the news, at that time, showed that the main topic was still regarding Covid-19 vaccines. The discussion focused on whether women in pregnancy or children are advised to take the vaccine. At the same time, worldwide attention has turned toward the situation in India. India was facing a second wave of Covid-19 with a rise in daily new cases which subsequently brought the national healthcare system to its limits [49–51].

In our analysis of the sentiment about AstraZeneca’s vaccine (shown in Fig. 7), we observed a significant change in sentiment around the first breakpoint, on 11/01/2021. At this time, there was significant discussion in the news about the company’s application for approval of its vaccine by the European Medicines Agency (EMA) [52, 53].

During the months of June and July 2021, and again in August 2022, the media focused extensively on the vaccination process. A study published in the British Medical Journal (BMJ) used real-world data to show that the AstraZeneca vaccine may be associated with a small increase in Immune Thrombocytopenic Purpura (ITP) [54]. As a result of the Thromboembolic events, on June 17, 2021, Australia restricted the use of the AstraZeneca vaccine to individuals over 60 years of age [55]. Vietnam has given the option for individuals who received the AstraZeneca vaccine to receive a second dose of an mRNA vaccine if they desire [56]. Moreover, a recent study in the United Kingdom discovered that the AstraZeneca and Pfizer vaccines provide a high level of protection against hospitalization due to the Delta variant of Covid-19 [57].

The proposed framework for sentiment analysis in this study offers several strengths. Firstly, its potential for application in contexts beyond Covid-19 demonstrates its versatility and adaptability, making it a valuable tool for analysing sentiment in various domains, assuming that there are similar basic characteristics, such as the existence of distinct categories, the limited text length according to the Twitter word limit and the availability of enough data for the training of the model. Secondly, the insights gained from analysing public sentiment towards the pandemic and its societal impacts can provide valuable information for informing public health policies, interventions and aiding decision-making processes. Additionally, the utilization of advanced techniques such as BERT and change point detection indicates a commitment to enhancing the accuracy of sentiment analysis and improving the reliability and validity of the findings. In addition, the framework proposed in this study has low computational cost and processing time, which may enhance its practical feasibility and efficiency in analysing sentiment in large datasets. To provide more specific details, the training time of BERT’s model was $\simeq 5$ minutes. The characterization of the 2, 157, 747 tweets, used for the time series, took $\simeq 35$ minutes and the detection of the changes took $\simeq 1.8$ seconds.

However, it is essential to acknowledge several limitations of the study. Firstly, the reliance on Twitter data as the source of sentiment analysis may introduce biases, as Twitter users may not be representative of the general population, and certain perspectives may be missed. Furthermore, the potential for bias in sentiment analysis itself is a critical concern, as accurately capturing the difference of human emotion through language alone is challenging and may result in skewed results. Moreover, the study’s cross-sectional design only captures a snapshot of sentiment at a specific time, which may not reflect changes in sentiment over time or across different populations, limiting the temporal and demographic generalizability of the findings. Lastly, another limitation of the proposed framework for sentiment analysis is its reliance on the entire time series data to identify the change point or breakpoint, which may not be possible for real-time analysis. This can potentially limit its applicability in situations where real-time monitoring of sentiment is required, such as during rapidly evolving events or crisis situations.

Overall, the proposed methodology could be applied to a use case scenario involving Covid-19 events to explore how exogenous factors, such as changes in health policies or regulations, have influenced public sentiment. By identifying sentiment change points, the methodology could provide insights into the impact of these policies on social opinion. Additionally, the analysis of sentiment change points could serve as a measure of public perception and acceptance of health policies. As the methodology analyses past events, it could be used to retrospectively evaluate the effectiveness of selected health policies in light of known events that have occurred during the pandemic. This highlights the potential of the methodology in evaluating the impact of health policies and regulations and understanding public sentiment and opinions in the context of the Covid-19 pandemic.

## Concluding remarks

The primary goal of this study was to propose a framework for sentiment analysis. This framework has been conducted on tweets with reference to Covid-19 within the year 2021. The most appropriate word representation model (BERT) has been selected through extensive experimentation in order to generate a time series of sentiment scores. Then, the identification of key periods of change in public attitudes has been detected by applying a change point detection algorithm. The visual investigation of word clouds, along with cross-referencing news reports from that time, gave us a deep insight into how people on Twitter felt about the pandemic and its effects on society. These findings highlight the potential usefulness of the methodology. Finally, the methodology, herein by identifying sentiment change points, provides insights into policy impact and public perception. It can also retrospectively evaluate policy effectiveness, highlighting its potential in understanding public sentiment during the pandemic. Future studies should concentrate on using online methods that could be explored as an alternative to offline methods for analysing real-time sentiment change detection as a situation or a trending topic is ongoing.

## Data Availability

The data used in this paper are publicly accessible and details are available in the references section.
